# Mobile Calibration Based on Laser Metrology and Approximation Networks

**DOI:** 10.3390/s100807681

**Published:** 2010-08-17

**Authors:** J. Apolinar Muñoz-Rodriguez

**Affiliations:** Centro de Investigaciones en Optica, Loma del Bosque 115, Col. Lomas del campestre, C.P. 37150, Leon, Guanajuato, Mexico; E-Mail: munoza@foton.cio.mx; Tel.: +477-441-4200; Fax: +477-441-4209.

**Keywords:** three-dimensional vision, laser line projection, mobile calibration, Bezier networks

## Abstract

A mobile calibration technique for three-dimensional vision is presented. In this method, vision parameters are computed automatically by approximation networks built based on the position of a camera and image processing of a laser line. The networks also perform three-dimensional visualization. In the proposed system, the setup geometry can be modified online, whereby an online re-calibration is performed based on data provided by the network and the required modifications of extrinsic and intrinsic parameters are thus determined, overcoming any calibration limitations caused by the modification procedure. The mobile calibration also avoids procedures involving references, which are used in traditional online re-calibration methods. The proposed mobile calibration thus improves the accuracy and performance of the three-dimensional vision because online data of calibrated references are not passed on to the vision system. This work represents a contribution to the field of online re-calibration, as verified by a comparison with the results based on lighting methods, which are calibrated and re-calibrated via perspective projection. Processing time is also studied.

## 1. Introduction

Nowadays in order to perform three-dimensional vision various lighting methods are used, such as fringe pattern projection, laser line and point projection, all of which require some form of calibration. Calibration for lighting methods is performed via perspective projection models [1]. In fringe projection, the calibration is performed based on calibrated references via perspective projection [2,3]. In this method, the three-dimensional vision is achieved by a phase detection algorithm. In line and point projection, the calibration is also achieved by perspective projection and the use of calibrated references [4,5], but here, the three-dimensional vision is performed by laser triangulation.

In the calibration and re-calibration of lighting methods, several methods based on perspective projection have been developed. One calibration method is performed by projecting a laser line on black and white rectangles [6,7]. The perspective projection is determined by matching the line to the known rectangles. A stereo calibration determines the perspective projection by matching a line of a grating and the use of epipolar geometry [8]. A paint brush method performs the calibration by projecting a line on two reference planes [9]. By detecting the line on these references, the perspective projection is determined via least squares. A lighting method performs the calibration based on the coordinates of a laser line [10]. Here, perspective projection is determined by transforming the laser line coordinates to real world coordinates. A zigzag method performs the calibration by detecting a laser line on zigzag references [11,12]. Based on these references, the perspective projection is obtained via a transformation matrix. A vision sensor performs the calibration by projecting a laser line on a reference plane [13–17]. In this case, the perspective projection is determined by detecting the line on this reference plane. A structure light system performs the calibration by projecting a pattern of spots on a reference plane [18,19]. The perspective projection is determined by detecting the spots on this plane. Another type of calibration is performed by projecting a spots pattern and a fringe pattern [20]. In this method, the perspective projection is determined by detecting the point-to-line correspondence on a plane. Re-calibration methods have also been implemented to change the vision parameters when the base setup is modified. One such re-calibration method is performed by detecting a pattern of lines on a reference plane to determine the perspective projection [21]. Self re-calibration methods have been implemented via plane-based homography [22–24], in which the perspective projection is determined by matching the light pattern on a reference plane.

Online re-calibration methods have also been developed to change the vision parameters during vision task [25–27]. In these methods, the perspective projection is determined by matching the light pattern on a reference plane. In the above mentioned techniques, the vision system does not provide the data to perform the re-calibration. Typically, these online re-calibration techniques are performed by detecting a light pattern on a reference. However, in several applications such references do not exist during the vision task, so the mentioned techniques are limited by the availability of light pattern references. To overcome these limitations, a re-calibration method without online references is necessary to facilitate online modifications of the setup geometry.

The proposed mobile calibration is performed by means of a Bezier network, which provides the data needed for online re-calibration, and laser line imaging. In this procedure, the camera orientation, focal distance, setup distances, pixel scale and image centre are determined. In addition three-dimensional vision is performed by the network via line shifting, whereby the network retrieves the surface depth and provides the data for the re-calibration when the setup geometry is modified online, the extrinsic and intrinsic parameters are thus re-calibrated online and the need for references is avoided. Consequently, the mobile calibration improves the performance and the accuracy of the online re-calibration. All this constitutes a contribution to the field of re-calibration of lighting methods. This contribution is elucidated by an evaluation based on the calibration and re-calibration of lighting methods. This evaluation is based on the root mean squared of error using a contact method as reference. Finally, the processing time to produce three-dimensional visualization is also determined.

## 2. Basic Theory

**Figure 1.**
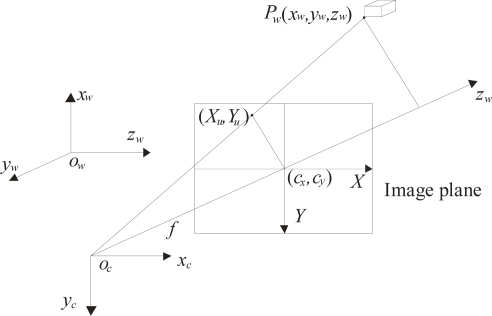
Geometry of the perspective projection model.

In lighting methods calibration is performed based on perspective projection [6–24]. This procedure is carried out by means of calibrated references and a transformation matrix. Typically, the perspective projection model is determined based in the geometry shown in Figure 1. In this geometry, a point *P_w_* = (*x_w_*, *y_w_*, *z_w_*) is transformed to the camera coordinates *P_c_* = (*x_c_*, *y_c_*, *z_c_*) by *P_c_* = **R**·*Pw* + ***t***. Where ***R*** is the rotation matrix and ***t*** is the translation vector. Here, the transformation *Pc* to the image coordinates (*X_u_*, *Y_u_*) is given by *X_u_* = *fx_c_*/*z_c_* and *Y_u_* = *fy_c_*/*z_c_* Considering radial distortion, the image coordinates are represented by *X_d_* + *D_x_* = *X_u_* and *Y_d_* + *D_y_* = *Y_u_*, where *D_x_* = *X_d_* (*δ*_1_*r*^2^ + *δ*_2_*r*^4^ + …), *D_y_* = *Y_d_* (*δ*_1_*r*^2^ + *δ*_2_*r*^4^ + …) and *r* = (*X_d_*^2^ + *Y_d_*^2^)^1/2^. In these expressions, *X_d_* and *Yd* are the distorted coordinates. The pixel coordinates are also converted into real coordinates by means of a scaling factor *η*. Thus, the parameters to be calibrated are the matrix ***R***, the vector ***t***, the focal length *f*, the distortion coefficient *δ_i_*, the image center (*c_x_*, *c_y_*) and the scaling factor *η*. This procedure is carried out by detecting calibrated references on a reference plane and use of a transformation matrix [6–27]. Then, the calibration data are passed to the vision system to perform three-dimensional visualization.

In several applications, the setup geometry is modified online to achieve good sensitivity and to avoid occlusions. In this case, a re-calibration is necessary for each modification [18,22]. In perspective projection, the translation vector ***t*** is the position vector from the *O_w_* to *O_c_*. This vector has components in the *x*-, *y*- and *z*-axes from the world coordinates *O_w_* to the camera coordinates *O_c_*. The distances of these components are determined in the initial calibration, but the components of vector ***t*** are modified when the camera is moved. In this case, these components are re-calibrated via calibrated references to perform the transformation from *P_w_* to *P_c_* [23]. The transformation *P_c_* = **R**·*Pw* + ***t*** to the coordinates (*X_u_*, *Y_u_*) should also be recomputed. However, in several applications calibrated references do not exist during the three-dimensional vision task, so established online re-calibration methods are limited by the availability of known references. To overcome these limitations, a re-calibration method without online references should be implemented.

**Figure 2.**
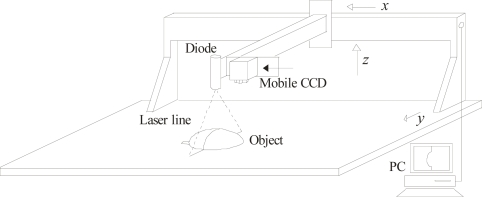
Mobile setup to perform the three-dimensional visualization.

In the proposed mobile calibration, a Bezier network provides the data to perform the online re-calibration and three-dimensional visualization based on a mobile setup and image processing of a laser line. The mobile setup to perform the three-dimensional vision is shown in Figure 2. This arrangement includes an electromechanical device, a CCD camera, a laser line projector and a computer to process the data. In this setup, the laser line is projected perpendicularly on the surface and the CCD image plane is aligned parallel to the reference plane. In this geometry, the laser line reflected to the CCD camera forms an angle that varies according to the position of the reference plane in the *z*-axis. The orientation of the CCD camera and the laser line orientation are fixed. The alignment of the camera and laser line are described in Section 3. The electromechanical device moves the laser and the camera in the *x*-axis, *y*-axis and *z*-axis. In addition the camera can also be moved toward the laser diode along in the *x*-axis.

**Figure 3.**
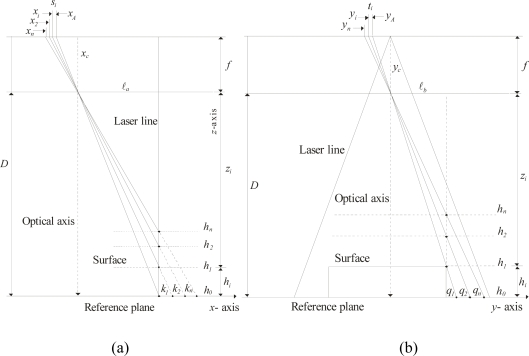
**(a)** Geometry of an image plane parallel to the reference plane in *x*-axis. **(b)** Geometry of an image plane parallel to the reference plane in *y*-axis.

In this system, a network computes the surface depth based on the line position. The geometry of this relationship is shown in Figure 3(a). In this geometry, the *x*-axis and *y*-axis are located on the reference plane and the *z*-axis is located perpendicularly to the reference plane. The focal length *f* is the distance between the lens and the image plane. The image center is indicated by *x_c_* on the *x*-axis. The distance between the laser line and the optical axis is indicated by ℓ*_a_*. The surface depth is indicated by *h_i_* and *z_i_* is the distance between the lens and the object surface. The distance from the lens to the reference plane is defined by *D* = *h_i_* + *z_i_*. In the proposed setup, the distance ℓ*_a_* and *D* can be modified during the visualization procedure. The laser line coordinates are indicated in the *y*-axis based on the geometry shown in Figure 3(b). In this geometry, a point *q_i_* of the laser line in the *y*-axis is indicated by *y_i_* in the image plane. Thus, the laser line coordinates are determined by *q_i_* = *D η* (*y_c_* − *y_i_*)/*η f*. In this expression *y_i_* is the image row and the parameters *D*, *f*, *η*, *y_c_* are deduced during the mobile calibration, which is described in Section 4. In perspective projection, the surface depth is computed by *z_i_* = (*f*·ℓ*_a_)/*(*x_c_* − *x_i_*) [1], based on the calibrated *f* and ℓ*_a_*. In the proposed model, the surface depth is computed based on the line shifting in the image plane. When the laser line is projected on a surface *h_i_*, the line position is moved from *x_A_* to *x_i_* in the image plane. In this case, the line shifting *s_i_* is directly proportional to the surface depth *h_i_*. This line shifting is described by following expression:

(1) $$s_{i}=x_{A}-x_{i}$$

To compute the shift, the line position *x_A_* and *x_i_* are detected in the image. To carry this out, the intensity maximum is measured in each row of the image. Then, first and second derivatives are computed to obtain the maximum. To detect the maximum, the pixels are approximated to a continuous function by means of Bezier curves [28]. In this case, the pixels are represented by (*x*_0_*, I*_0_), (*x*_1_, *I*_1_),......, (*x_n_, I_n_*), where *x_i_* is the pixel position, *I_i_* is the pixel intensity and *n* is the pixel number. The Bezier curves are described by:

(2) $$\begin{array}{llll} P\left(u\right)=\sum_{i=0}^{n}\left(\begin{array}{c} n\\ i \end{array}\right){\left(1-u\right)}^{n-i}u^{i}p_{i}, & & \left(\begin{array}{c} n\\ i \end{array}\right)=\frac{n!}{i!\left(n-i\right)!}, & 0\leq u\leq 1 \end{array}$$

By applying the definition of Equation (2), two equations are obtained, one for *x* and one for *I*:

(3) $$x\left(u\right)=\left(\begin{array}{c} n\\ 0 \end{array}\right)\;{\left(1-u\right)}^{n}u^{0}x_{0}+\left(\begin{array}{c} n\\ 1 \end{array}\right)\;{\left(1-u\right)}^{n-1}u\;x_{1}+\ldots \ldots .+\left(\begin{array}{c} n\\ n \end{array}\right)\;{\left(1-u\right)}^{0}u^{n}x_{n,}0\leq u\leq 1$$

(4) $$I\left(u\right)=\left(\begin{array}{c} n\\ 0 \end{array}\right)\;{\left(1-u\right)}^{n}u^{0}I_{0}+\left(\begin{array}{c} n\\ 1 \end{array}\right)\;{\left(1-u\right)}^{n-1}u\;I_{1}+\ldots \ldots +\left(\begin{array}{c} n\\ n \end{array}\right)\;{\left(1-u\right)}^{0}u^{n}I_{n,}0\leq u\leq 1$$

**Figure 4.**
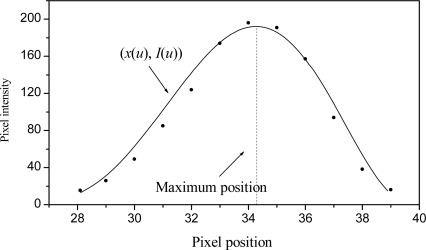
Fitted pixels to a continuous function by Bezier curves.

Equation (3) represents the pixel position and Equation (4) represents the pixel intensity. Based on these equations, a continuous function is fitted from the pixels shown in Figure 4. To carry this out, the positions *x_0_*, *x_1_*, *x_2_*,…, *x_n_*, are substituted into Equation (3) and the intensities *I_0_*, *I_1_*, *I_2_*,..,*I_n_*, are substituted into Equation (4). These two equations are evaluated in the interval 0 ≤ *u* ≤ 1 to fit the curve shown in Figure 4. The result of this curve is a concave function. Therefore, the second derivative *I’*(*u*) is positive and the peak is a global maximum. In this manner, the maximum is computed based on the first derivative *I’*(*u*) = 0 [29]. To find the derivative *I’*(*u*) = 0, the bisection method is applied [29]. The Bezier function is defined in the interval 0 ≤ *u* ≤ 1, so the initial value is defined by *u_i_* = 0 and the final value is indicated by *u_f_* = 1. Then, the middle point is computed by u* = (*u_i_* + *u_f_*)/2 to find a value *u* that converges to the expression *I*′(*u*) = 0. Next the first derivative *I*′(*u*) is evaluated in middle point u*. If the derivative *I’*(*u* = u*) is positive, then *u_i_* = u*. If the derivative *I’*(*u* = u*) is negative, then *u_f_* = u*. The next middle point u* is obtained from the last pair of values *u_i_* and *u_f_*. These steps are repeated until *I*′(*u*) = 0 is found based on some set tolerance value. The value *u* = u* where *I*′(*u*) = 0 is substituted into Equation (3) to determine the position of the intensity maximum *x*(*u*). The result is *x*(*u*) = 34.274 and the laser line position is *x_i_* = 34.274 pixels, as shown in Figure 4. Thus, the laser line position is detected. The Bezier network to perform the three-dimensional vision and calibration is described in Section 3.

## 3. Network Structure for Depth Contouring

**Figure 5.**
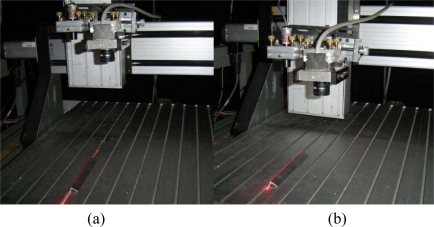
**(a)** Laser line aligned on a peak reference in *y*-axis. **(b)** Setup at different reference plane from the initial configuration.

The three-dimensional vision is performed by a Bezier network based on the line shifting. This network is built based on an image plane parallel to the reference plane in the *x*-axis and *y*-axis. Based on the geometry of Figure 3(a), the laser line is perpendicular to the reference plane in the *x*-axis. In this case, the line position along the *x*-axis is constant for any surface depth *h_i_*. By means of this criterion, the laser line is aligned perpendicularly to the *x*-axis. To carry this out, the laser line is projected on a peak reference along the *y*-axis. Figure 5(a) shows the laser line aligned in the *y*-axis on the peak reference. Then, the reference plane is moved in the *z*-axis, as shown in Figure 5(b). By rotating the laser diode in 0.0896 degree steps, the laser line is positioned in the reference for any depth *h_i_* of the reference plane. By image processing, the reference position and the line position are detected in each displacement of depth *h_i_*. In this case, the line position is the same in the *x*-axis for any position of the reference plane. Thus, a perpendicular laser line to the reference plane in the *x*-axis is achieved. Now, the image plane is aligned parallel to the reference plane in the *x*-axis. Based on the setup geometry of Figure 3(a), the term (*k_i_*/*h_i_*) = [*ℓ_a_*/(*D* − *h_i_*)] = [(*ηx_c_* − *ηx_i_*)/*η f*] is obtained. In this term, *η* is the scale factor in millimeters, *f* is in pixels and (*x_c_* − *x_i_*) = *s_i_* + (*x_c_* − *x_A_*). Thus, the following expression for the line shifting is obtained:

(5) $$k_{i}=\frac{\eta (x_{c}-x_{i})h_{i}}{\eta f}=\frac{[(x_{c}-x_{A})+s_{i}]h_{i}}{f}$$

In Equation (5) *f*, *x*_c_, and *x_A_* are constants. In this case, a linear *h_i_* produces a linear *s_i_*. Conversely, a linear *s_i_* produces a linear *k_i_*. Therefore d*k*/d*s*, the derivative of *k_i_* with respect to *s_i_*, is a constant. Another camera orientation is an optical axis not perpendicular to the reference plane. In this case, a linear *s_i_* does not produce a linear *k_i_* and the derivative d*k*/d*s* is not a constant.

The camera orientation along the *y*-axis is performed based on the geometry of Figure 3(b). In this geometry, a line pattern is moved in steps *y_i_* in the image plane in the *y*-axis based on depth *h_i_*. In this geometry, the optical axis is perpendicular to the reference plane and the term (*q_i_*/*h_i_*) = [*ℓ_b_*/(*D* − *h_i_*)] = [*η* (*y_c_* − *y_i_*)/*η f*] is obtained. Here, the position of the pattern shifting is computed by *t_i_* = (*y_0_* − *y_i_*) = (*y_c_* − *y_i_*) − (*y_c_* − *y_A_*). Thus, the following expression is obtained:

(6) $$q_{i}=\frac{\eta (y_{c}-y_{i})h_{i}}{\eta f}=\frac{[(y_{c}-y_{A})+t_{i}]h_{i}}{f}$$

**Figure 6.**
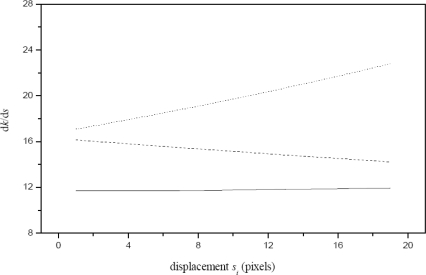
Derivative d*k*/d*s* for an optical axis perpendicular and not perpendicular to the *x*-axis.

In Equation (6) *f*, *y*_c_ and *y_A_* are constant. In this case, a linear *h_i_* produces a linear *t_i_*. A linear *t_i_* also produces a linear *q_i_*, so the derivative d*q*/d*t* is a constant. In this manner, the camera orientation is defined by the d*k*/d*s* = constant and d*q*/d*t* = constant. Due to the distortion, these derivatives are not exactly a constant, but they may be considered constant. For the camera orientation in the *x*-axis, the reference plane is moved from *h_0_* to *h*_1_, *h*_2_, *h*_3_,…, *h_n_* by means of the electromechanical device. For each depth *h_i_*, the line position *x_i_* is computed by the procedure described in Section 2. Then, the shifting *s_i_* is computed via Equation (1), the derivative d*k*/d*s* is computed and it is evaluated with respect to the derivative d*k_1_*/d*s*. If d*k*/d*s* is bigger than d*k_1_*/d*s* the camera is rotated to the right in steps of 0.0896 degree. Again, the reference plane is moved from *h_0_* to *h*_1_, *h*_2_, *h*_3_,…, *h_n_* and the derivative is computed. If d*k*/d*s* is less than d*k_1_*/d*s* the camera is moved in the opposite direction. The rotation to the left and right is repeated until the minimum error of derivative d*k*/d*s* respect to d*k_1_*/d*s* is found. In this case, the derivative d*k*/d*s* is not exactly a constant, but it is close to constant. This criterion is illustrated by the derivative shown by the solid line in Figure 6, where the dashed line represents d*k*/d*s* of an optical axis aligned at an angle smaller than 90°. The dotted line is d*k*/d*s* of an optical axis aligned at an angle greater han 90°.

**Figure 7.**
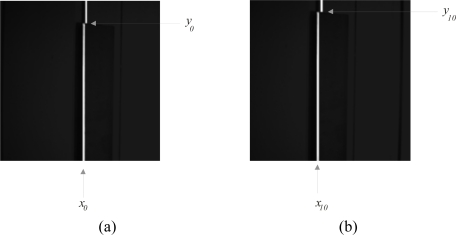
**(a)** Laser line on the reference plane. **(b)** Laser line at 25.4 mm from the reference plane.

For the *y*-axis camera orientation, the d*q*/d*t* is computed. To carry this out, the position *t_i_* is computed in the image plane based on *h_i_*. This is done by detecting the corner of the laser line in the *y*-axis via edge detection. Figure 7(a) shows the corner position *y_0_* of the line in the *y*-axis at the reference plane *h*_0_. Then, the reference plane is moved in the *z*-axis and the corner is detected to obtain *y_1_*. This procedure is repeated to obtain *y*_2_, *y*_3_,…, *y_n_*. Figure 7(b) shows the corner position *y*_10_ of the line in *y*-axis at the reference plane *h*_10_. Based on these data, *t_i_* and the derivative d*q*/d*t* are computed. Then, this derivative is evaluated with respect to the derivative d*q_1_*/d*t*. The camera is rotated in the *y*-axis to the right or the left in the same manner as it was rotated in the orientation in the *x*-axis.

**Figure 8.**
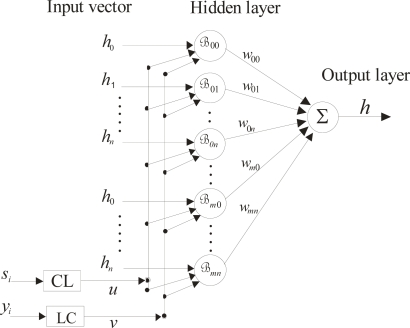
Structure of the Bezier network.

The derivative d*q*/d*t* obtained in this procedure is not exactly a constant, but again it is close to being a constant. Therefore, the camera parallel to the reference plane is defined when the d*k*/d*s* and d*q*/d*t* are very close to a constant according to a tolerance. In this manner, the image plane has been aligned parallel to the reference plane. From here, the alignment of the laser line is achieved perpendicular to the reference plane and the camera is fixed. Based on an image plane parallel to the reference plane, the network is built. To carry this out, the data of the camera alignment *h_i_* and *s_i_* are used. The structure of the proposed network is shown in Figure 8. This network consists of an input vector, two parametric inputs, a hidden layer and an output layer. Each layer of the network is constructed as follows: the input includes the depth *h_i_*, the line shifting *s_i_* and the parametric values (*u*, *v*). The depth data *h_0_*, *h*_1_, *h*_2_,…, *h_n_* and the line shifting data *s_0_*, *s*_1_, *s*_2_,…, *s_n_* are obtained in the camera alignment by moving the reference plane in the z-axis. Thus, the line shifting *s_i_* is directly proportional to the surface depth *h_i_*. In this case, the line shifting is represented by a parametric value *u* by the next linear combination (LC):

(7) $$u=a_{0}+a_{1}s$$

where *a*_0_ and *a*_1_ are constants to be determined. By means of two values *s_i_* and its respective value *u*, Equation (7) is determined. The Bezier curves are defined in the interval 0 ≤ *u* ≤ 1. Therefore, *u* = 0 for the first line shifting and *u* = 1 for the last shifting *s_n_*. Substituting these values in Equation (7), two equations with two unknown constants are obtained. Solving these equations, *a*_0_ and *a*_1_ are determined. Thus, for each shifting *s_i_*, a value *u* is computed via Equation (7).

The coordinate *y_i_* corresponds to each row of the laser line image. This coordinate is represented by a parametric value *v* by the following expression:

(8) $$v=b_{0}+b_{1}y$$

where *b*_0_ and *b*_1_ are constants to be determined. Using two values *y_i_* and its respective *v*, Equation (8) is determined. Bezier curves are defined in the interval 0 ≤ *v* ≤ 1. In this case, *v* = 0 for *y_0_* and *v* = 1 for *y_n_*. Substituting these two values in Equation (8), two equations with two unknown constants are obtained. Solving these equations, *b*_0_ and *b*_1_ are determined. Thus, for each coordinate *y_i_*, a value *v* is computed via Equation (8). The hidden layer is built by a Bezier basis function, which is described by:

(9) $${ℬ}_{\mathrm{ij}}=B_{i}\left(u\right)\;B_{j}\left(v\right)$$

where $B_{i}\left(u\right)=\left(\begin{array}{c} n\\ i \end{array}\right)u^{i}{\left(1-u\right)}^{n-i}$, $B_{j}\left(v\right)=\left(\begin{array}{c} m\\ j \end{array}\right)u^{j}{\left(1-v\right)}^{m-j}$, $\left(\begin{array}{c} n\\ i \end{array}\right)=\frac{n!}{i!\left(n-i\right)!}$, $\left(\begin{array}{c} m\\ j \end{array}\right)=\frac{m!}{j!\left(m-j\right)!}$.

The output layer is obtained by the summation of the neurons, which are multiplied by a weight. Thus, the output response is the surface depth given by following expression:

(10) $$\begin{array}{cccccc} ℋh\left(u,v\right)=\sum_{i=0}^{n}\sum_{j=0}^{m}w_{\mathrm{ij}}h_{i}\;B_{i}\left(u\right)\;B_{j}\left(v\right), & & & 0\leq u\leq 1, & & 0\leq v\leq 1 \end{array}$$

where *w_ij_* are the weights, *h_i_* is the surface depth, *B_i_*(*u*) and *B_j_*(*v*) are the Bezier basis function represented by Equation (9). To construct the complete network Equation (10), the appropriate weights *w_ij_* should be determined. To carry this out, the network is being forced to produce the correct surface depth *h_i_*. This procedure is performed by an adjustment mechanism. Based on the reference data obtained in camera alignment, the initial depth *h_0_* = 0 mm, the line position in the image plane is *x_A_* = *x*_0_ and *s_0_* = 0. The line position *x_0_* in *x*-axis is shown in Figure 7(a). In the camera alignment, the reference plane is moved in the *z*-axis in steps of 2.54 mm. Thus, *h*_10_ = 25.40 mm and the line position correspond to *x_i_* = *x*_10_ which are shown in Figure 7(b). Here, the shifting is determined by *s*_10_ = *x_A_* − *x_i_*. Then, the *s_i_* and its coordinate *y_i_* are converted to values (*u*, *v*) via Equation (7) and Equation (8), respectively. Then, the depth *h_i_* and its coordinates (*u*, *v*) are substituted in Equation (10) to obtain an output H(*u*, *v*), thus giving the following system of equations:

(11) $$\begin{array}{c} H\left(u=0,v=0\right)=h_{0}=w_{00}h_{0}B_{0}\left(u\right)B_{0}\left(v\right)+w_{01}h_{0}B_{0}\left(u\right)B_{1}\left(v\right)+,\ldots ,+w_{0m}h_{0}B_{0}\left(u\right)B_{m}\left(v\right)+,\ldots ,\\ +w_{10}h_{1}B_{1}\left(u\right)B_{0}\left(v\right)+,\ldots ,+w_{1m}h_{1}B_{1}\left(u\right)B_{m}\left(v\right)+,\ldots ,+w_{\mathrm{nm}}h_{n}B_{n}\left(u\right)B_{m}\left(v\right)\\ H\left(u,v\right)=h_{1}=w_{00}h_{0}B_{0}\left(u\right)B_{0}\left(v\right)+w_{01}h_{0}B_{0}\left(u\right)B_{1}\left(v\right)+,\ldots ,+w_{0m}h_{0}B_{0}\left(u\right)B_{m}\left(v\right)+,\ldots ,\\ +w_{10}h_{1}B_{1}\left(u\right)B_{0}\left(v\right)+,\ldots ,+w_{1m}h_{1}B_{1}\left(u\right)B_{m}\left(v\right)+,\ldots ,+w_{\mathrm{nm}}h_{n}B_{n}\left(u\right)B_{m}\left(v\right)\\ \begin{array}{ccccccccc} \vdots & & \vdots & & \vdots & & & & \vdots \end{array}\\ H\left(u=0,v=1\right)=h_{n}=w_{00}h_{0}B_{0}\left(u\right)B_{0}\left(v\right)+w_{01}h_{1}B_{0}\left(u\right)B_{1}\left(v\right)+,\ldots ,+w_{0n}h_{n}B_{0}\left(u\right)B_{n}\left(v\right)+\\ ,\ldots ,+w_{m0}h_{0}B_{m}\left(u\right)B_{0}\left(v\right)+,\ldots ,+w_{\mathrm{mn}}h_{n}B_{m}\left(u\right)B_{n}\left(v\right)\\ \begin{array}{ccccccccc} \vdots & & \vdots & & \vdots & & & & \vdots \end{array}\\ H\left(u=1,v=0\right)=h_{0}=w_{00}h_{0}B_{0}\left(u\right)B_{0}\left(v\right)+w_{01}h_{0}B_{0}\left(u\right)B_{1}\left(v\right)+,\ldots ,+w_{0m}h_{0}B_{0}\left(u\right)B_{m}\left(v\right)+,\ldots ,\\ +w_{10}h_{1}B_{1}\left(u\right)B_{0}\left(v\right)+,\ldots ,+w_{1m}h_{1}B_{1}\left(u\right)B_{m}\left(v\right)+,\ldots ,+w_{\mathrm{nm}}h_{n}B_{n}\left(u\right)B_{m}\left(v\right)\\ \begin{array}{ccccccccc} \vdots & & \vdots & & \vdots & & & & \vdots \end{array}\\ H\left(u=1,v=1\right)=h_{n}=w_{00}h_{0}B_{0}\left(u\right)B_{0}\left(v\right)+w_{01}h_{0}B_{0}\left(u\right)B_{1}\left(v\right)+,\ldots ,+w_{0m}h_{0}B_{0}\left(u\right)B_{m}\left(v\right)+,\ldots ,\\ +w_{10}h_{1}B_{1}\left(u\right)B_{0}\left(v\right)+,\ldots ,+w_{1m}h_{1}B_{1}\left(u\right)B_{m}\left(v\right)+,\ldots ,+w_{\mathrm{nm}}h_{n}B_{n}\left(u\right)B_{m}\left(v\right) \end{array}$$

This linear system of Equation (11) can be represented as:

(12) $$\begin{array}{c} H_{00}=w_{00}\beta_{0,0}+w_{01}\beta_{0,1}+,\ldots ,+w_{\mathrm{nm}}\beta_{0,nm}\\ H_{01}=w_{00}\beta_{1,0}+w_{01}\beta_{1,1}+,\ldots ,+w_{\mathrm{nm}}\beta_{1,nm}\\ \vdots \;\;\;\;\;\;\;\vdots \;\;\;\;\;\vdots \;\;\;.....\vdots \\ H_{\mathrm{nm}}=w_{00}\beta_{\mathrm{mn},0}+w_{01}\beta_{\mathrm{mn},1}+,\ldots ,+w_{\mathrm{nm}}\beta_{\mathrm{nm},nm} \end{array}$$

This equation can be rewritten in matrix form as ***β* W** = **H**. Thus, the linear system is represented by the following matrix:

(13) $$\left[\begin{array}{cccc} \beta_{0,0} & \beta_{0,1} & \beta_{0,2} & ....\;\beta_{0,nm}\\ \beta_{1,0} & \beta_{1,1} & \beta_{1,2} & ....\;\beta_{1,nm}\\ \vdots & \vdots & \vdots & \vdots \\ \beta_{\mathrm{nm},0} & \beta_{\mathrm{nm},1} & \beta_{\mathrm{nm},2} & ....\;\beta_{\mathrm{nm},nm} \end{array}\right]\left[\begin{array}{c} w_{00}\\ w_{01}\\ \vdots \\ w_{\mathrm{nm}} \end{array}\right]=\left[\begin{array}{c} H_{00}\\ H_{01}\\ \vdots \\ H_{\mathrm{nm}} \end{array}\right]$$

**Figure 9.**
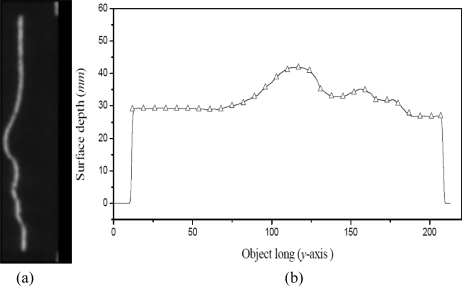
**(a)** Laser line projected on a surface. **(b)** Surface depth computed by the network form the laser line.

The linear system Equation (13) is solved by the Chelosky method and the weights *w_ij_* are determined. In this manner, the Bezier network H(*u*,*v*) has been completed. The result of this network is a model that computes the surface depth *h*(*x*, *y*) via line shifting. The network is applied to the laser line shown in Figure 9(a) to obtain the surface depth. To carry this out, the shifting *s_i_* is detected in each image row *y_i_*. Then, the shifting *s_i_* and its coordinate *y_i_* are converted to a value (*u*, *v*), respectively. Then, these values are substituted in the network Equation (10) to compute the surface depth shown in Figure 9(b). In this figure, the symbol “Δ” is the data provided by a coordinate measurement machine (CMM).

To determine the accuracy, the network data are compared with the data provided by the CMM. The accuracy is computed based on a root means squared error (*rms*) [30] by:

(14) $$\mathrm{rms}=\sqrt{\frac{1}{n}\sum_{i=1}^{n}{\left({\mathrm{ho}}_{i}-{\mathrm{hc}}_{i}\right)}^{2}}$$

where *ho_i_* is the data provided by the CMM, *hc_i_* is the calculated data by the network and *n* is the number of data. For the data shown in Figure 9(b), the error is a *rms* = 0.148 mm. The depth resolution is deduced by the detection of the minimum line shifting *s_i_*. In this case, the network is built using the minimum and maximum *s_i_* at distance ℓ*_a_*. For this configuration, a shifting *s_i_* = 0.38 pixels is detected from the reference plane. Based on this shifting, the network computes a depth *h* = 0.28 mm. Thus, the small details around *h* = 0.28 mm can be detected and the network sensibility has been determined. The calibration of vision parameters based on the network is described in Section 4.

## 4. Parameters of the Vision System

In the lighting methods, the calibration is performed based on perspective projection [6–22]. In this model, the extrinsic and intrinsic parameters are determined based on calibrated references. Thus, the matrix ***R***, vector ***t***, focal length *f*, distortion *δ_i_*, image center (*c_x_*, *c_y_*) and the scale factor *η* are calibrated. Typically, these lighting systems do not provide the data needed to perform the re-calibration. Any time the setup is modified a re-calibration should be applied. This procedure provides the vision system with the ability to change the intrinsic and extrinsic parameters. This was tested when the camera position is modified. In this case, the components of vector ***t*** are changed and the distances from the origin of the world coordinates *O_w_* to the camera coordinates *O_c_* should be re-calibrated. Recently, self re-calibration and online re-calibration have been developed to change the vision parameters [22–27]. In these methods, the data for online re-calibration are determined by detecting a light pattern on calibrated references. This kind of re-calibration is suitable when the references exist during the vision task, but in several applications such references do not exist. In this case, the online re-calibration cannot be completed due to the lack of references and an online re-calibration without references is thus necessary to overcome this.

In the proposed mobile calibration, the data for online re-calibration is provided the Bezier network and laser line imaging, thus avoiding the need for pattern references. The proposed vision system can be moved in the *x*-, *y*- and *z*-axes. In addition the camera can be moved toward the laser line. Here, the setup geometry is modified when the camera is moved in the *z*-axis. The geometry is also modified when the camera is moved toward the laser line. In this case, a mobile calibration is applied to perform the online re-calibration. This procedure is performed based on the setup geometry shown in Figure 3(a). Here, the calibration is performed based on the camera being parallel to the reference plane. The triangulation of this geometry is described by the expression of Equation (5). Considering radial distortion, the line position is defined by *x_A_* = *X_A_* + *δx_A_* and *x_i_* *= X_i_* + *δx_i_*, respectively, where *X_A_* and *X_i_* are the undistorted image coordinates and the distortion is indicated by *δx_A_* and *δx_i_*, respectively. Thus, the line shifting is defined by *S_i_* = (*x_c_* − *X_i_*) − (*x_c_* − *X_A_*). Therefore, the projection *k_i_* Equation (5) is rewritten as:

(15) $$K_{i}=\frac{\eta (x_{c}-X_{i})h_{i}}{\eta f}=\frac{[(x_{c}-X_{A})+S_{i}]h_{i}}{f}$$

The distortion can be described from the terms of Equation (5) and Equation (15) by the following expression:

(16) $$\begin{array}{c} h_{i}=\frac{{f\;K}_{i}}{\eta [(x_{c}-X_{A})+S_{i}]}=\frac{{\mathrm{fk}}_{i}}{\eta [(x_{c}-x_{A})+s_{i}]}\\ \frac{K_{i}}{\left[(x_{c}-x_{A}+\delta x_{A})+(s_{i}+\delta x_{i}-\delta x_{A})\right]}=\frac{k_{i}}{\left[(x_{c}-x_{A})+s_{i}\right]} \end{array}$$

From this equation, the distortion *δx_i_* is described by:

(17) $${\delta x}_{i}=\frac{K_{i}}{k_{i}}(x_{c}-x_{A}+s_{i})+(x_{A}-x_{c}-s_{i})$$

The distortion can be determined from the terms of the line shifting *S_i_* = (*x_c_* − *X_i_*) − (*x_c_* − *X_A_*) and *s_i_* = (*x_A_* − *x_i_*). Here, the first line shifting *s_1_* is defined without distortion. In this manner, *S_i_* = i* *s_1_*, *K_i_* = i* *k*_1_ and d*K_i_*/d*S* = d*k_1_*/d*s* for *i* = 1, 2,…, *n*. Based on these criteria, the undistorted shifting is described by the term i* *s_1_* = (*x_c_* − *x_i_* + *δx_i_*) − (*x_c_* − *x_A_* + *δx_A_*) = (*x_A_* − *x_i_*) + (*δx_i_* − *δx_A_*). Thus, the distortion is obtained by *δx_i_* = i* *s_1_* − (*x_A_* − *x_1_*) + *δx_A_*. From this expression, the first line shifting is defined without distortion. Therefore, *δx_A_* = 0, *δx_1_* = 0, *S_1_* = *s_1_* and the distortion *δx_1_* = i* *s_1_* − (*x_A_* − *x_1_*). Thus, the distortion is defined by:

(18) $${\delta x}_{i}=i*s_{1}-\left(x_{A}-x_{i}\right)+{\delta x}_{A}=i*s_{1}-\left(x_{A}-x_{i}\right)\;\text{for}\;i=2,3,\ldots ,n$$

The *y*-axis distortion is determined based on the setup geometry shown in Figure 3(b). The triangulation of this geometry is described by the expression of Equation (6). Considering radial distortion, *y_A_* = *Y_A_* + *δy_A_* and *y_i_* = *Y_i_* + *δy_i_*, where *Y_A_* and *Y_i_* are the undistorted image coordinate and the distortion is indicated by *δy_A_* and *δy_i_*, respectively. Thus, the pattern shifting is defined by *T_i_* = (*y_c_* − *Y_A_*) − (*y_c_* − *Y_i_*). In this manner, the projection *q_i_* Equation (6) is rewritten as:

(19) $$Q_{i}=\frac{\eta (y_{c}-Y_{i})h_{i}}{\eta f}=\frac{[(y_{c}-Y_{A})+T_{i}]h_{i}}{f}$$

The procedure to determine the distortion in *x*-axis is applied to find the distortion *δy_i_*. Thus, the distortion in *y*-axis is defined by:

(20) $${\delta y}_{i}=i*t_{1}-\left(y_{A}-y_{i}\right)+{\delta y}_{A}=i*t_{1}-\left(y_{A}-y_{i}\right)\;\text{for}\;i=2,3,\ldots ,n$$

In this manner, the distortion has been deduced. Based on an image plane parallel to the reference plane, the vision parameters are deduced. This procedure is carried out based on the setup geometry Figure 3(a), which is described by:

(21) $$\frac{\ell_{a}}{D-h_{i}}=\frac{\eta (x_{c}-X_{i})}{f}=\frac{\eta [(x_{c}-X_{A})+S_{i})]}{f}$$

In this equation, the constants *D*, *ℓ_a_*, *f*, are in millimeters, *x_c_* is in pixels and *η* is the scale factor. To determine these parameters, Equation (21) is rewritten as the following system of equations:

(22) $$\begin{array}{c} h_{0}=\frac{f\ell_{a}}{\eta \left(x_{c}-X_{A}+S_{0}\right)}+D\\ h_{1}=\frac{f\ell_{a}}{\eta \left(x_{c}-X_{A}+S_{1}\right)}+D\\ h_{2}=\frac{f\ell_{a}}{\eta \left(x_{c}-X_{A}+S_{2}\right)}+D\\ h_{3}=\frac{f\ell_{a}}{\eta \left(x_{c}-X_{A}+S_{3}\right)}+D\\ h_{4}=\frac{f\ell_{a}}{\eta \left(x_{c}-X_{A}+S_{4}\right)}+D \end{array}$$

The values *h*_0_, *h*_1_,…, *h*_4_, are computed by the network based on *s*_0_, *s*_1_,…, *s*_4_. The values *X_A_* and *S_i_* are computed using the known *s_i_*, *δx_A_* and *δx_i_*, then these values are substituted in Equation (22) to solve the system of equations and thus determine the constants *D*, *ℓ_a_*, *f*, *η*, and *x_c_*. The coordinate *y_c_* is computed based on the geometry of Figure 3(b). Here, the parameters *η*, *T_i_* = *I* × *t_1_*, *Y_i_* are known and *T_i_* = *η*(*y_c_* − *Y_i+1_*) − *η*(*y_c_* − *Y_1_*). Thus, *y_c_* is determined by the following system of equations:

(23) $$\begin{array}{c} T_{1}=\eta \left(y_{c}-Y_{2}\right)-\eta \left(y_{c}-Y_{1}\right)\\ T_{2}=\eta \left(y_{c}-Y_{3}\right)-\eta \left(y_{c}-Y_{1}\right) \end{array}$$

**Figure 10.**
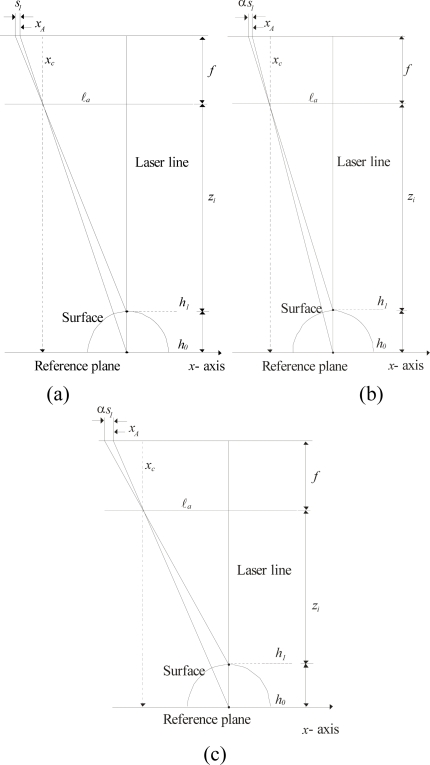
**(a)** Initial geometric configuration. **(b)** Geometry of the camera moved toward the laser line in *x*-axis. **(c)** Geometry of the camera moved toward the object surface in z-axis.

The values *T*_1_, *T*_2_, *Y_1_*, *Y_2_* and *Y_3_* are collected from the camera orientation in the *y*-axis. These values are substituted in Equation (23) to solve the system and thus the value *y_c_* is determined. The laser line coordinates are determined based on the parameters *D, η*, *y_c_*, and *f*. In this case, *y_i_* are the image coordinates of the laser line in the *y*-axis. Based on the geometry of Figure 3(b), the coordinates of laser line in the *y*-axis are determined by the term *q_i_* = *D η*(*y_c_* − *y_i_*)/*η f*. In this manner, the vision parameters have been determined by the data provided by network and image processing. The mobile setup generates online geometric modifications, giving the system the ability to overcome occlusions and attain high sensibility. Typically, the extrinsic parameters are re-calibrated when the camera changes position [31]. In the reported methods, the online re-calibration depends on the availability of calibrated references [24–29]. The proposed mobile calibration avoids the use of references for online re-calibration. In the proposed vision system the setup geometry [Figure 10(a)], is modified when the camera is moved toward the laser line along the *x*-axis, as seen in Figure 10(b). The setup geometry is also modified when the camera is moved in the *z*-axis [Figure 10(c)]. In these cases, the line shifting magnitude should be re-calibrated online. The distance to the object surface should also be recalibrated online when the camera is moved in the *z*-axis. This procedure is carried out by computing the line shifting factor *α*. From the initial configuration [Figure 10(a)], the expression *tanθ*1 = *η f*/(*X_A_* − *x_c_*) *η* describes the line position. From the geometry shown in Figure 10(b), the expression *tanθ*_2_ = *η f*/(*αX_A_* − *x_c_*) *η* describes the line position. In these expressions, *f*, *x_c_* are known from the initial calibration and *αX_A_* is a line position in the new configuration [Figure 10(b)]. Here, the position *αX_A_* is defined as the smaller distance obtained from the term (*αX_j_* − *x_c_*).

In this case, the term *αX_j_* is the line position in each row of the image in *y*-axis and the *j*-index is row number of the image. Based on these data, the angles *θ*_1_ and *θ*_2_ are computed. Thus, the expression (*X_A_* − *x_c_*) *tanθ*_1_ = (*αX_A_* − *x_c_*) *tanθ*_2_ is obtained. Therefore, the factor *α* is determined by:

(24) $$\alpha =\frac{\left(X_{A}-x_{c}\right)\;\text{tan}\;\theta_{1}}{X_{A}\;\text{tan}\;\theta_{2}}+\frac{x_{c}}{X_{A}}$$

Then, the shifting is divided by the factor *α* in the modified configuration. The shift *s_i_* is re-calibrated online and thus processed by the network to obtain the surface *h_i_*. In this manner, the vision system provides an online re-calibration of line position and the line shifting and the need for calibrated references is avoided. In the perspective projection, the extrinsic and intrinsic parameters change when the camera is moved, therefore components of the translation vector ***t*** should be re-calibrated to perform the transformation *P_c_* = **R**·*Pw* + ***t***. Methods such as object-based and plane-based homography have been applied to perform the online re-calibration of vision parameters. Object-based methods perform the re-calibration based on the known marks on the object [32]. Homography methods perform the re-calibration based on the detection of light pattern references [22–24]. These reported methods are limited when the references do not exist during the vision task.

**Figure 11.**
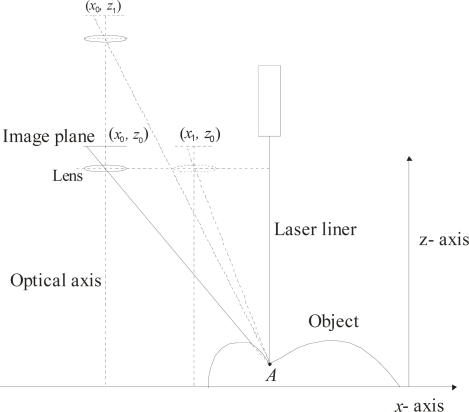
Geometry of an occlusion in the initial configuration.

The contribution of the mobile calibration when an occlusion appears during the vision task was also studied. Typically, occlusions appear due to the surface variation and a big distance *ℓ_a_*. This criterion is studied based on the geometry shown in Figure 11, in which the point *A* is occluded at the initial configuration when the image plane is placed at (*x*_0_, *z*_0_). Here, an occlusion is detected when the width of the line is less than three pixels.

To avoid occlusions, the camera is moved away from the surface at (*x*_0_, *z*_1_) in the *z*-axis or toward the laser line at (*x*_1_, *z*_0_) in the *x*-axis. Here, an occlusion is detected when the width of the line is smaller than three pixels along the laser line in the *y*-axis. This criterion is established according to a threshold value. When an occlusion appears, the surface scanning is stopped. Then, the camera is moved by one 1.27 mm step toward the laser line. In this camera position, the width of the laser line is evaluated. If the width of laser line is less than three pixels, the camera is moved one step toward the laser line. This procedure is repeated until to the width of the laser line is greater than three pixels. Then, the scanning is continued with the new camera position and the occluded region is thus detected based on a complete laser line. In this case, the shift magnitude is different from the initial configuration, so the shift should be re-calibrated based on the factor *α* via Equation (24). Then, the shift *s_i_* is processed by the network to compute the object surface. The three-dimensional vision and calibration accuracy are described in Section 5.

## 5. Experimental Results

**Figure 12.**
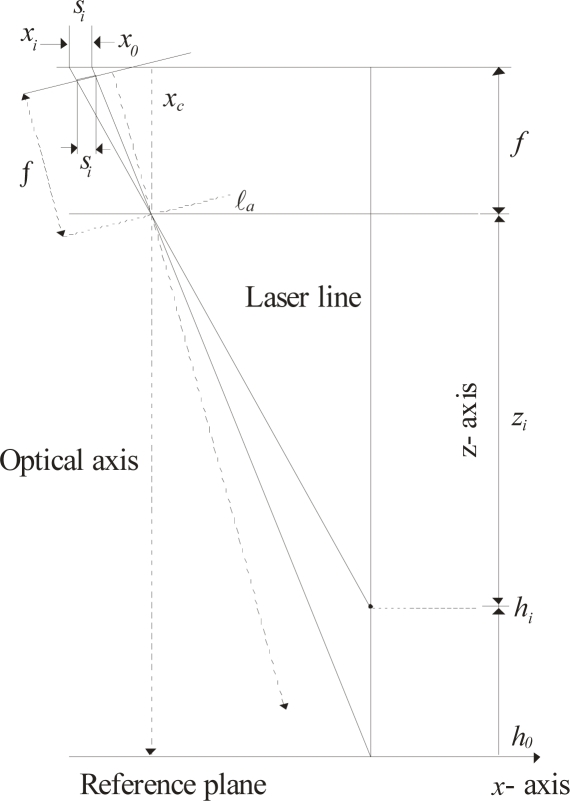
Geometry of an image plane parallel and not parallel to the reference plane.

The proposed mobile calibration is performed based on Bezier networks and laser line imaging. This automatic technique avoids calibrated references and physical measurements. Here, the three-dimensional visualization is performed by laser scanning in 1.27 mm steps along the *x*-axis. The scanning movement is provided by the electromechanical device, whose minimum step is 0.0245 mm. The positioning accuracy is 0.00147 mm and the positioning repeatability is a standard deviation ± 0.001 mm. The laser line is captured and digitized in 256 gray levels. From each image, the network computes the surface contour based on the line shift *s_i_*. The depth resolution provided by the network is around of 0.28 mm. This resolution corresponds to the distances *ℓ_a_* = 174.8321 mm and *D* = 380.4126 mm. In this case, the depth resolution varies according to the distances *ℓ_a_* and *D*. The depth resolution is increased when *ℓ_a_* is increased and *D* is decreased. On the other hand, depth resolution is decreased when *ℓ_a_* is decreased and *D* is increased. The image plane parallel to the reference plane provides better sensitivity for a laser line perpendicular to the reference plane. This criterion is proven by the geometry of Figure 12. In this case, the image plane parallel to the reference plane provides a bigger line shift than the rotated image plane, but the rotated image plane provides better sensitivity when the laser line is aligned at an angle. Therefore, the image plane is fixed parallel to the reference plane and perpendicular to the laser line, thus affording the best sensitivity. When the setup geometry is modified, a mobile calibration is performed based on the data provided by the network and image processing. This procedure provides high sensitivity and avoids occlusions. This was verified by the three-dimensional visualization of some surfaces with small and big details.

**Figure 13.**
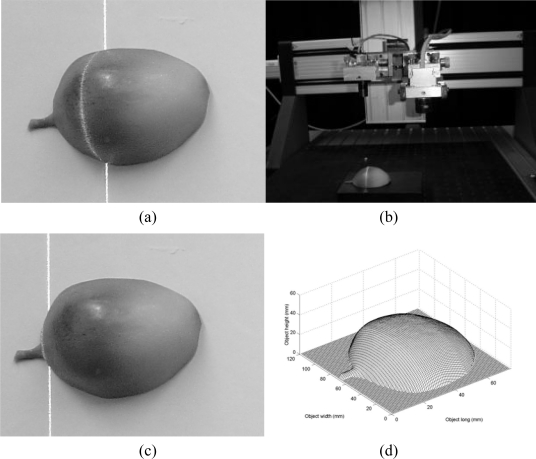
**(a)** Plastic fruit to be profiled. **(b)** Mobile setup for three-dimensional vision. **(c)** Occlusion based on the broken line. **(d)** Three-dimensional shape of the plastic fruit.

The first test of the mobile calibration is the three-dimensional visualization of a plastic fruit, shown in Figure 13(a). To carry this out, the fruit is scanned by the vision system as shown in Figure 13(b). In this procedure, a set of images is captured by the CCD camera. From each image, the line shift *s_i_* is detected via Equation (1) and converted to a value *u* via Equation (7). In addition, the coordinate *y_i_* of the laser line is converted to a value *v* via Equation (8). Then, the network computes a transverse section of the object by substituting the values (*u*,*v*) in Equation (10). In this scanning, an occlusion is detected by the broken line shown in Figure 13(c). To avoid the occlusion, the camera is moved toward the laser line. In doing so, the initial geometry has been modified, so the line shift should be re-calibrated online based on the factor *α* via Equation (24). To carry this out, the line position *αX_j_* is detected in each row of the images in the *y*-axis. In this case, the term *αX_j_* is the line position and the *j*-index is the row number of the image in the *y*-axis. Here, the position *αX_A_* is the smaller distance from the term (*αX_j_*−*x_c_*). Then, the factor *α* is computed and the line shift is divided by this factor to achieve the online re-calibration and the re-calibrated shift *s_i_* is substituted in the network Equation (10) to compute the surface *h_i_*. The whole surface is reconstructed from all depth data provided by the network. Fifty six images were processed to obtain the plastic fruit shown in Figure 13(d), where the scale of this figure is in mm.

To know the accuracy, a root means squared error (*rms*) is computed via Equation (14). To do this the plastic surface is measured by the CMM. This procedure is performed by measuring a transverse section of the object in 8.0 mm steps along the *y*-axis. The transverse section corresponds to the position where the laser line was projected on the *x*-axis. Then, the network computes the surface depth of this transverse section. Forty six transverse sections are measured by the CMM and by the network in steps of 1.27 mm. In this case, the data obtained from the CMM are *n* = 420. Then, error is computed and the result is a *rms* = 0.142 mm.

**Figure 14.**
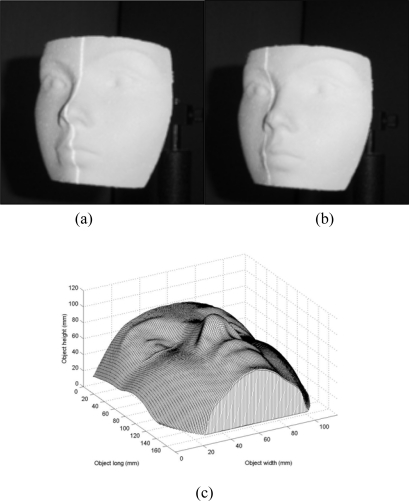
**(a)** Dummy face to be profiled. **(b)** Occlusion based on the broken line. **(c)** Three-dimensional shape of the dummy face.

The second test of the mobile calibration is the vision of a dummy face shown in Figure 14(a). To carry this out, the dummy face is scanned by the vision system. In this scanning, no reference plane exists. This was established based on the absence of a line at the end and beginning of the image in the *y*-axis. Here, the shifting magnitude is different from the initial configuration, so the line shift should be re-calibrated online. In this case, the line position *αX_j_* is detected in each row of the image in the *y*-axis. From these line positions, the position *αX_A_* is determined as the minimum distance from the term (*αX_j_* − *x_c_*). Then, the factor *α* is computed via Equation (24) and the line shift is divided by this factor *α* to achieve the online re-calibration and the re-calibrated shift *s_i_* is converted to a value *u*. In addtion, the coordinate *y_i_* of the laser line is converted to a value *v*. Then, the network computes the data *h_i_* of a transverse section of the object by substituting the values (*u*,*v*) in Equation (10).

When the object is scanned, an occlusion is detected based on the broken line shown in Figure 14(b). To avoid the occlusion, the camera is moved toward the laser line. Since the geometry and the shift magnitude have been modified the mobile calibration performs a second online re-calibration to achieve the three-dimensional vision. To carry it out, the line position *αX_j_* is detected in each row of the image in the *y*-axis for the new configuration. From these line positions, the *αX_A_* is determined as the minimum from the term (*αX_j_* − *x_c_*). Then, the factor *α* is computed via Equation (24). and the shifting is divided by this factor *α* to achieve the online re-calibration. This re-calibrated shifting *s_i_* is converted to a value *u*. The coordinate *y_i_* of the laser line is also converted to a value *v*. Then, the network computes the surface depth *h_i_* by substituting the values (*u*,*v*) in Equation (10). In this manner, the whole surface is obtained by all data provided by the network. One hundred and sixteen images are processed to obtain the complete dummy face shown in Figure 14(c). The scale of this figure is in mm. To know the accuracy, the *rms* is computed via Equation (14). To do this, the transverse sections of the dummy face are measured by the CMM and by the network. In this case, sixty two transverse sections are measured by the CMM at 1.00 mm in the *x*-axis to perform the evaluation. From this procedure, the obtained data are *n* = 1,400. Then, the error is computed and the result is a *rms* = 0.155 mm.

**Figure 15.**
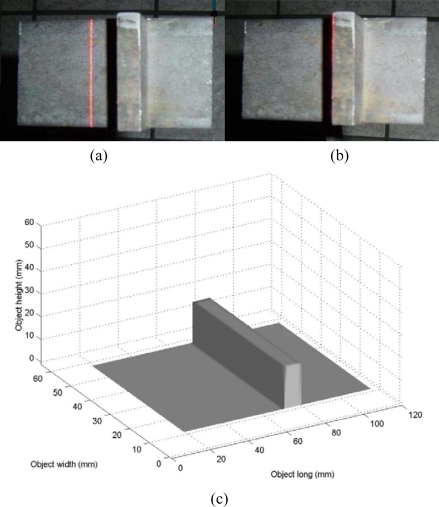
**(a)** Metallic piece to be profiled. **(b)** Occlusion based on the broken line. **(c)** Three-dimensional shape of the metallic piece.

The third test of the mobile calibration is the visualization of the flat surface of a metallic piece shown in Figure 15(a). To carry this out, the metallic piece is scanned by the vision system. In this scanning, no reference plane exists during the vision procedure. This is stated based on the lack of a line at the end and beginning of the *y*-axis in the image. Since the line shift magnitude is different from the initial configuration, mobile calibration is applied to achieve the online re-calibration of the line shift. To do so, the line position *αX_j_* is detected in each row of the image in the *y*-axis. In this case, the line position *X_j_* is the same along the laser line and *X_0_* = *X_1_* = *X_2_*,…, = *X_n_* and the minimum distance from the term (*αX_j_* − *x_c_*) can be obtained by means of *αX_0_*. Therefore, the reference position is determined by *αX_A_* = *αX_0_*. Then, the factor *α* is computed via Equation (24) abd the line shift is divided by the factor *α* to achieve the online re-calibration. Then, the re-calibrated shift *s_i_* is converted to a value *u* via Equation (7). The coordinate *y_i_* of the laser line is also converted to a value *v* via Equation (8). Then, the network computes the depth *h_i_* by substituting the values (*u*,*v*) in Equation (10). In the scanning, an occlusion is detected based on the missing line shown in Figure 15(b). To avoid the occlusion, the camera is moved toward the laser line. Here, the geometry and the shift magnitude have been modified, so the mobile calibration performs a second online re-calibration to achieve the three-dimensional visualization. To carry it out, the line position *αX_j_* is detected in each row of the image in the *y*-axis. In this case, position *αX_j_* is the same along the laser line. Again, the reference position *αX_A_* is determined by *αX_A_* = *αX_0_*. Then, the factor *α* is computed via Equation (24) and the shift is divided by this factor *α* to achieve the on-line recalibration and the re-calibrated shift *s_i_* is then converted to a value *u*. The coordinate *y_i_* of the laser line is also converted to a value *v* and the network computes the surface depth *h_i_* by substituting the values (*u*,*v*) in Equation (10). In this manner, the whole surface is obtained from data provided by the network. Fifty eight images were processed to obtain the metallic piece shown in Figure 14(c). The scale of this figure is in mm. To know the accuracy, the metallic piece was measured by the CMM. Then, the *rms* is computed using the data provided by the CMM and by the network. In this procedure, the error is computed using *n* = 1,400 and error is a *rms* = 0.155 mm.

The value *n* has a great influence in the precision of the calculated error. To determine if *n* is according to the desired precision, the confidence level [33] is calculated by:

(25) $$n={\left(z_{\alpha }\frac{\sigma_{x}}{e}\right)}^{2}$$

where *z_α_* is the desired confidence, *e* is the error expressed in percentage, and *σ_x_* is standard deviation. Therefore, the confidence level based on the data *n* is described by:

(26) $$z_{\alpha }=\frac{e}{\sigma_{x}}\sqrt{n}$$

To know if the chosen *n* is according to the desired confidence, Equation (26) is applied. The desired confidence is 95%, which corresponds to *z_α_* = 1.96 according to the confidence table [33]. The average of the dummy face height is 44.32 mm. Therefore, the *rms* is an error of 0.0035, which represents a 0.35% error. To determine the error precision, the confidence is calculated via Equation (25) for *n* = 1,400, *e* = 0.35 and a standard deviation of 6.204. The result is *z_α_* = 2.110, which indicates a confidence level over 95%. The confidence levels for the plastic fruit and for the metallic piece are also greater than 95%.

The accuracy provided by the calibration via Bezier networks for the three-dimensional vision is less than 1%. For comparison, the best accuracy of the calibration and online recalibration are mentioned as follows: a stereo calibration via perspective projection for face profiling reports an error over 1% [8]. A calibration method based on perspective projection and least squares for a laser range scanner reports an error over 1% [9]. A calibration method based on perspective projection and the invariance of double cross-ratio of the cross-points reports an error of 2% [16]. A self plane-based homography re-calibration method reports an error of 1% [23]. An online re-calibration method based on homography and reference plane reports an error of 1.5% [27]. These results indicate that the proposed mobile calibration provides better accuracy, based on its error of less than 1%. The mobile calibration also avoids external procedures and the need for calibrated references to perform the online re-calibration. The resolution provided by proposed technique is good, according to the calibration methods based on perspective projection using similar distances to our setup [6–27]. In these reports, a paint brush laser range scanner reports 0.57 mm as the best resolution [9]. The measurement range of the proposed technique is in the interval between 0.3 mm and 280.60 mm. According to the above mentioned techniques [6–27], the measurement range of the proposed mobile setup is good.

The computer used in this vision system is a 1.8 GHz PC. The capture velocity of the camera is 34 fps. The electromechanical device is moved at 34 steps per second. Each image of laser line is processed by the network in 0.010 s. The shape of the dummy face was reconstructed in 3.88 s, the metallic piece was reconstructed in 3.22 s and the plastic fruit was profiled in 2.59 s. This processing time is good compared to the lighting methods based on perspective projection. To demonstrate this, the processing time of the fast techniques is given as follows: for a paint brush laser range scanner, the reported time to reconstruct a single view is 15 s [9]. In implementation and experimental studies on fast object modeling based on multiple structured stripes, the reported time to reconstruct a single view of the object is 10 s [17]. These results indicate that the proposed mobile system provides a fast three-dimensional visualization. In this procedure, physical measurements and calibrated references are avoided to perform online re-calibration of the vision parameters and the distances of the geometry of the setup are not used to compute the surface depth, so the proposed mobile calibration performed online using data provided by the network and image processing is easier than the online re-calibration techniques based on references and perspective projection. In this manner, the vision system achieves good repeatability, corresponding to a standard deviation ± 0.01 mm.

## 6. Conclusions

A mobile calibration technique for three-dimensional vision has been presented. In this technique, a Bezier network provides the data needed to perform the mobile calibration via image processing. The network also computes the object surface based on the mobile setup. The setup geometry can thus be modified online and the network provides the online re-calibration to needed to accurately perform the three-dimensional visualization. The automatic calibration avoids the need for physical measurements and calibrated references, which are used in the lighting methods based on perspective projection. This improves the performance of the vision system and the accuracy of the three-dimensional visualization. The ability to detect the laser line with a sub-pixel resolution has been achieved by using Bezier curves, and the image processing is achieved with few operations. With the automatic calibration, good repeatability is achieved in each three-dimensional visualization procedure. The technique described here should provide a valuable tool for industrial inspection and reverse engineering tasks.
